# Radix Astragalus Polysaccharide Accelerates Angiogenesis by Activating AKT/eNOS to Promote Nerve Regeneration and Functional Recovery

**DOI:** 10.3389/fphar.2022.838647

**Published:** 2022-04-01

**Authors:** Geyi Zhang, Jinsheng Huang, Shuang Hao, Jingchao Zhang, Nan Zhou

**Affiliations:** ^1^ Department of Orthopedics, The First Affiliated Hospital of Zhengzhou University, Zhengzhou, China; ^2^ Department of Cardiac Surgery, The First Affiliated Hospital of Zhengzhou University, Zhengzhou, China

**Keywords:** astragalus polysaccharide, angiogenesis, axonal regeneration, functional recovery, peripheral nerve injury

## Abstract

Peripheral nerve injury (PNI) results in loss of neural control and severe disabilities in patients. Promoting functional nerve recovery by accelerating angiogenesis is a promising neuroprotective treatment strategy. Here, we identified a bioactive Radix Astragalus polysaccharide (RAP) extracted from traditional Chinese medicine (TCM) as a potent enhancer of axonal regeneration and remyelination. Notably, RAP promoted functional recovery and delayed gastrocnemius muscle atrophy in a rat model of sciatic nerve crush injury. Further, RAP treatment may induce angiogenesis *in vivo*. Moreover, our *in vitro* results showed that RAP promotes endothelial cell (EC) migration and tube formation. Altogether, our results show that RAP can enhance functional recovery by accelerating angiogenesis, which was probably related to the activation of AKT/eNOS signaling pathway, thereby providing a polysaccharide-based therapeutic strategy for PNI.

## Introduction

Peripheral nerve injury (PNI), which is associated with a poor prognosis and a high disability rate, results in loss of sensory, motor, and autonomic functions and neuropathic pain (Robinson, 2000; Jones et al., 2016). Although peripheral nerves have a strong regenerative capacity, nerve regeneration can lack specificity. Axonal regeneration is disordered, and the target organ is incorrectly reconnected, resulting in poor functional recovery (Allodi et al., 2012). Currently, autologous nerve grafting remains the gold standard in PNI treatment (Conforti et al., 2014). However, the strategy also has many limitations, and only about 80% of patients achieve full functional recovery (Sun et al., 2018; Houshyar et al., 2019). Therefore, novel methods to promote functional recovery are urgently needed.

Vascularization is one of the great challenges for neural reconstruction, and the role of intraneural angiogenesis has often been ignored (Xia and Lv, 2018; Saffari et al., 2020). In vertebrates, vascularization is essential for nerve regeneration, and angiogenesis precedes neurogenesis while sharing the same structural principles and molecular mechanisms as those responsible for nervous system wiring (Zacchigna et al., 2008). Recent studies have found that blood vessels can act as cues for nascent axons, directing axonal regeneration (Cattin et al., 2015). After PNI, nerve regeneration is dependent on local microcirculation and requires an increased oxygen and nutrient supply (Fu et al., 2007). In addition, it has been shown that 2D nanomaterials can promote peripheral neurogenesis by facilitating angiogenesis (Yan et al., 2021). In this case, adequate vascularization is not only important for providing essential nutrients for nerve regeneration but also for removing degenerative and necrotic substances from the trauma site, thus maintaining the viability of the newly regenerated nerve (Muangsanit et al., 2018; Zhao et al., 2019). Therefore, we hypothesized that functional recovery might be promoted by accelerated angiogenesis.

Radix Astragali (*Astragalus membranaceus* or “Huang Qi”) is one of the most important “Qi” tonic herbs in traditional Chinese medicine (TCM) and is commonly used for enhancing the immune system and strengthening the physique (Auyeung et al., 2016; Li et al., 2018). A water soluble polysaccharide with an average molecular weight of 1334 kDa was named RAP, which was composed of Rha, Ara, Glc, Gal, and GalA in a molar ratio of 0.03:1.00:0.27:0.36:0.30, has been shown to have various biological effects, such as immunomodulation and anti-tumor, anti-inflammatory, and antioxidant activities (Song et al., 2008; Yin et al., 2012). Previous studies showed that RAP induced the secretion of interleukin-1β (IL-1β), tumor necrosis factor-α (TNF-α), IL-10, IL-12p40, and granulocyte-macrophage colony-stimulating factor (GM-CSF) in human mononuclear cells (Yin et al., 2012). Further research showed that RAP can induce macrophage polarization toward the M1 phenotype and that its anti-tumor effect is related to its immunomodulatory effect on macrophages (Wei et al., 2016; Wei et al., 2019). In addition, RAP significantly reduced the toxic effects of taxol on immune cells (Bao et al., 2018). RAP was shown to enhance recovery after cyclophosphamide-induced myelosuppression by increasing FOS expression (Bao et al., 2021). However, the effect of RAP on functional recovery after PNI has not been investigated.

In this study, we used RAP intraneural injection to treat sciatic nerve crush injuries for the first time. We hypothesized that RAP would promote functional nerve recovery by accelerating angiogenesis. To test this, we evaluated the effect of RAP on endothelial cell (EC) migration and tube formation *in vitro*. The results showed that the RAP (100 μg/ml) significantly increased EC migration and tube formation, which may be associated with the activation of AKT/eNOS signaling pathway. In addition, RAP was a potent enhancer of ordered axonal regeneration and remyelination. Notably, RAP promoted functional recovery and delayed gastrocnemius atrophy in a rat model of sciatic nerve crush injury. Moreover, the neuroprotective effect of RAP may be due to the activation of AKT/eNOS signaling pathways to accelerate angiogenesis. Overall, these results show the great potential of RAP in promoting sciatic nerve regeneration.

## Materials and Methods

### RAP Preparation

RAP isolation and purification have been described previously (Yin et al., 2012). Air-dried astragalus was pounded into powder, extracted twice with boiling water, filtered, and concentrated under reduced pressure. The solution was precipitated with anhydrous ethanol. The precipitate was dissolved again and deproteinized. Then, it was dialyzed with distilled water, lyophilized, and the lyophilized material was retained. The lyophilized material was dissolved in distilled water, separated on a Hiload 26/60 Superdex-200 column, and eluted with water. The eluate was fractionated, dialyzed, and finally lyophilized to obtain RAP.

### Scratch Wound Migration Assay

Human umbilical vein endothelial cells (HUVECs) were purchased from Procell Life Science&Technology Co., Ltd (Wuhan, China). HUVECs were seeded on 6-well plates and incubated overnight. After reaching a fusion rate of approximately 90%, the cell monolayer was scraped in a cross shape using a 100 µl pipette tip (at time 0). The cell monolayers were then washed three times with phosphate-buffered saline (PBS) to ensure that no cells remained in the scratched area, and the cells were incubated in RAP-containing medium for 24 h. Images were captured with a phase-contrast microscope (Olympus, Tokyo, Japan). Scratch areas were measured at five random locations using ImageJ software. Wound healing rate = (0 h scratch area—24 h scratch area)/0 h scratch area×100%.

### Transwell Chamber Migration Assay

Cell migration was measured using a transwell chamber with 8-μm filter inserts (Corning, USA) without Matrigel. The upper chamber was filled with 200 μl of cell suspension at 5 × 10^4^ cells/mL. The lower chamber was filled with 700 μl of Dulbecco’s Modified Eagle’s Medium (DMEM) containing different concentrations of RAP. Transwell chambers were removed after 24 h and cells on the upper side of chambers were removed with cotton swabs. Then, chambers were washed with PBS, and cells on the lower side were fixed with 4% paraformaldehyde for 30 min, stained with 1% crystal violet for 30 min, and then washed with PBS and air-dried. Each chamber was observed under a phase-contrast microscope, and images of five randomly selected fields of view were acquired, and the cells were counted using ImageJ software.

### Capillary Network Formation Assay

HUVECs (2×10^4^) were resuspended in 100 μl of DMEM containing different concentrations of RAP, and the cell suspension was added to a 96-well plate precoated with 50 µl of Matrigel and incubated for 4 h. The medium was discarded, and images were acquired using a phase-contrast microscope. The number of branch points was analyzed using ImageJ software.

### RNA-Sequencing and Bioinformatics Methods

RNA was extracted by the Trizol and assayed for RNA integrity and total amount with 2,100 bioanalyzer (Agilent, CA, USA). Library construction will be performed using RAP treatment for 24 h and control RNA samples (n = 3), and then sequenced on Illumina HiSeq 6,000(Illumina, CA, USA). RNA sequencing technology was provided by Novogene (Beijing, China). Differential expression analysis was performed using the DESeq2 R package (1.20.0) for two groups. Padj ≤0.05 and |log2 (foldchange)| ≥ 0 were set as the threshold for differential expression. Gene Ontology (GO) and Kyoto Encyclopedia of Genes and Genomes (KEGG) enrichment analysis of differentially expressed genes (DEGs) was implemented by the clusterProfiler R package (3.8.1). Protein-protein interaction (PPI) network analyses with Cytoscape software (3.9.0).

### Western Blot

Total HUVEC protein was extracted using RIPA Lysis Buffer containing protease and phosphatase inhibitors. Protein concentration was measured using the BCA protein assay kit (Solarbio, Beijing, China). Total proteins were separated in 7.5% SDS-polyacrylamide gels and transferred to PVDF membranes (Bio-Rad, Hercules, CA, USA). Primary antibodies and corresponding dilutions were as follows: AKT (1:10000, Abcam, ab179463), p-AKT (1:5000, Abcam, Ab81283), eNOS (1:1000, Abcam, ab199956), p-eNOS (1:1000, Abcam, ab215717), PI3K (1:1000, Abcam, ab191606), p-PI3K (1:1000, #17366, CST). Secondary antibodies and corresponding dilutions were as follows: Goat Anti-Mouse IgG (1:5000, Solarbio, SE131), Goat Anti-rabbit IgG (1:5000, Solarbio, SE134). β-actin (1:1000, Solarbio, K200058M) was used as an internal control. Images are collected using Amersham Imager 600 (GE, CT, USA) and analyzed by ImageJ software. The experiment was repeated three times.

### Animal Models and Drug Delivery

Adult male Sprague-Dawley rats (200–250 g) were obtained from SJA Laboratory Animal Company (Hunan, China). The living conditions and experimental procedures conformed to the National Institutes of Health (NIH) Guide Concerning the Care and Use of Laboratory Animals. All animal experiments were approved by the Animal Experimentation Ethics Committee of Zhengzhou University. Five rats per cage were housed under controlled environmental conditions of temperature (23 ± 2°C), humidity (35–60%), and a 12:12-h light-dark cycle.

The animal model of nerve crush injuries has been described previously (Suzuki et al., 2017). Briefly, after anesthesia with 10% chloral hydrate (3 mg/kg), the rats were fixed in a prone position on the operating table, and the right sciatic nerve was exposed in a sterile environment. Then, the nerve was crushed 3 times for 10 s at 30 s intervals, at 5 mm from the bifurcation of the sciatic nerve using Dumont No. Five forceps, until the nerve had a translucent band, which was marked with a 10–0 suture. Rats were randomly divided into three groups: PNI group, RAP group, and sham group (n = 5 for each group).

The PNI group and the RAP treatment group received intraneural injections of 5 µl PBS or RAP, respectively. The needle was left in place for 5 min after the injection to prevent drug leakage. The sham-operated group underwent the same procedure without sciatic nerve injury. Finally, the wound was sutured. After 28 days, all rats were sacrificed, and the right sciatic nerve was collected and analyzed.

### Walking Track Analysis

To assess functional recovery, walking track analysis was performed on all rats postoperatively at 7, 14, and 28 days as previously described (Matsuoka et al., 2018). The rats were placed in a long box (60 cm × 7.5 cm × 7.5 cm) with the end protected from light and the bottom lined with flat white paper. Red ink was applied to both hind limbs of the rats, and they were allowed to walk freely in the box. Rats were trained to walk in the box without stopping for the duration of the experiment. Sciatic Functional Index (SFI) was calculated with the following formula. SFI = -38.3×[(EPL-NPL)/NPL]+109.5 [(ETS-NTS)/NTS]+[(EIT-NIT)/NIT]-8.8, where 0 indicates normal neurological function and -100 indicates complete loss of function (Feng and Yuan, 2015).

### Morphological and Histological Analysis

For morphological analysis, collected sciatic nerves or gastrocnemius muscles were fixed overnight in 4% paraformaldehyde (PFA), then dehydrated in gradient grade ethanol, and embedded in paraffin wax. After paraffin embedding, longitudinal or transverse sections (5 μm) were de-waxed and hydrated. Hematoxylin and eosin (HE), and Masson staining was performed according to the manufacturer’s protocol. Finally, slides were fixed with neutral resin and capped. Images of stained sections were acquired with a light microscope (Olympus, Japan).

### Immunofluorescence Staining

Immunofluorescence staining was performed according to the manufacturer’s protocols. Primary antibodies and corresponding dilutions were as follows: NF-200 (1:200, CST, 2836S), S100β (1:200, Abcam, ab52642), CD31 (1:200, Abcam, ab182981), CD34 (1:200, Abcam, ab81289), VEGFR (1:200, Abcam, ab2349), AKT (1:100, Abcam, ab179463), p-AKT (1:100, Abcam, ab192623), eNOS (1:100, ThermoFisher, PA1-037), p-eNOS (1:200, ThermoFisher, PA1-037). Secondary antibodies and corresponding dilutions were as follows: FITC-labeled goat anti-rabbit (1:200, Goodbio, GB22303), CY3-labeled goat anti-mouse (1:300, Goodbio, GB21301), CY3-labeled goat anti-rabbit (1:300, Goodbio, GB21303), AlexaFluor®488-labeled goat anti-rabbit (1:400, Servicebio, GB25303). Nuclei were labeled with 4′6-Diamidino-2-phenylindole-dihydrochloride (DAPI, Servicebio, G1012). Fluorescence images were acquired with fluorescence microscope (Olympus, Japan). Fluorescence intensity was analyzed with ImageJ software.

### Electron Microscopy

The sciatic nerve on the injured side was collected, trimmed to a 1 mm^3^ block of tissue, and fixed in 2.5% glutaraldehyde. The tissue was then immersed in 1% osmic acid for 2 h and dehydrated with acetone. Subsequently, the samples were encapsulated in epoxy resin and oven-dried. Samples were sectioned in 1.5-μm semithin sections and 60-nm ultrathin sections. The 1.5-μm semithin sections were used for toluidine blue (TB) staining, and images were acquired with a light microscope (Olympus, Japan). The 60-nm ultrathin sections were stained with 2% uranyl acetate-2.6% lead citrate solution, and images were acquired with a projection electron microscope (HITACHI, Japan). G-ratio and myelin sheath thickness were measured with ImageJ software.

### Statistical Analysis

Statistical analysis was performed using GraphPad Prism 8.0 (GraphPad Software, La Jolla, CA, USA). The results are presented as the means ± SD. One-way ANOVA was used for comparisons within multiple groups, and a two-tailed unpaired Student’s t-test was used for comparisons between two groups. Statistical significance was defined as *p* < 0.05.

## Results

### RAP Promoted EC Migration

**FIGURE 1:**
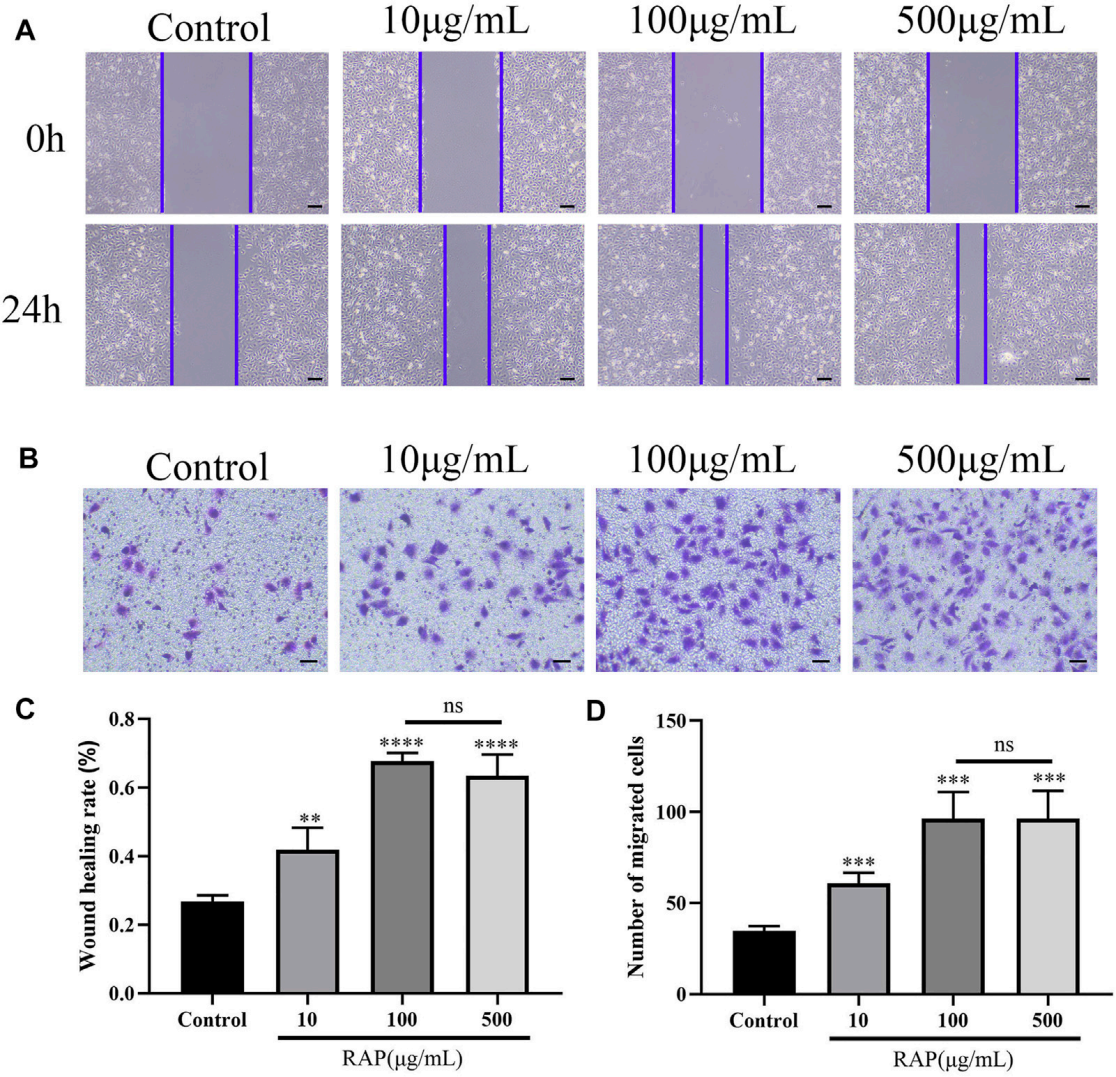
RAP promoted EC migration **(A)** Scratch wound migration assay *in vitro*. Representative images of migrating ECs treated with different concentrations of RAP for 24 h (40× amplification, scale bar = 200 μm); **(B)** Transwell chamber migration assay *in vitro.* Representative images of vertical migration of ECs treated with different concentrations of RAP for 24 h (100× amplification, scale bar = 100 μm); **(C)** Wound healing rate of ECs treated with different concentrations of RAP for 24 h; **(D)** The number of migrating ECs treated with different concentrations of RAP for 24 h. Data are presented as means ± SD. **p < 0.01, ***p < 0.001, ****p < 0.0001 compared with the control group.

Scratch wound and transwell chamber migration assays were performed to assess the effect of different concentrations of RAP on EC migratory capacity. When RAP concentration was ≤100 μg/ml, the scratch healing rate (Figures 1A,C, *p* < 0.05) and the number of migrating cells (Figures 1B,D, *p* < 0.05) were significantly increased compared with the control group. However, no significant differences were observed at RAP concentrations between 100 and 500 μg/ml (*p* > 0.05). In summary, RAP at concentrations ≤100 μg/ml increased EC migration in a concentration-dependent manner, with the strongest effect at a concentration of 100 μg/ml. The effect was similar at concentrations of 100 μg/ml and 500 μg/ml.

### RAP Promoted EC Tube Formation

**FIGURE 2:**
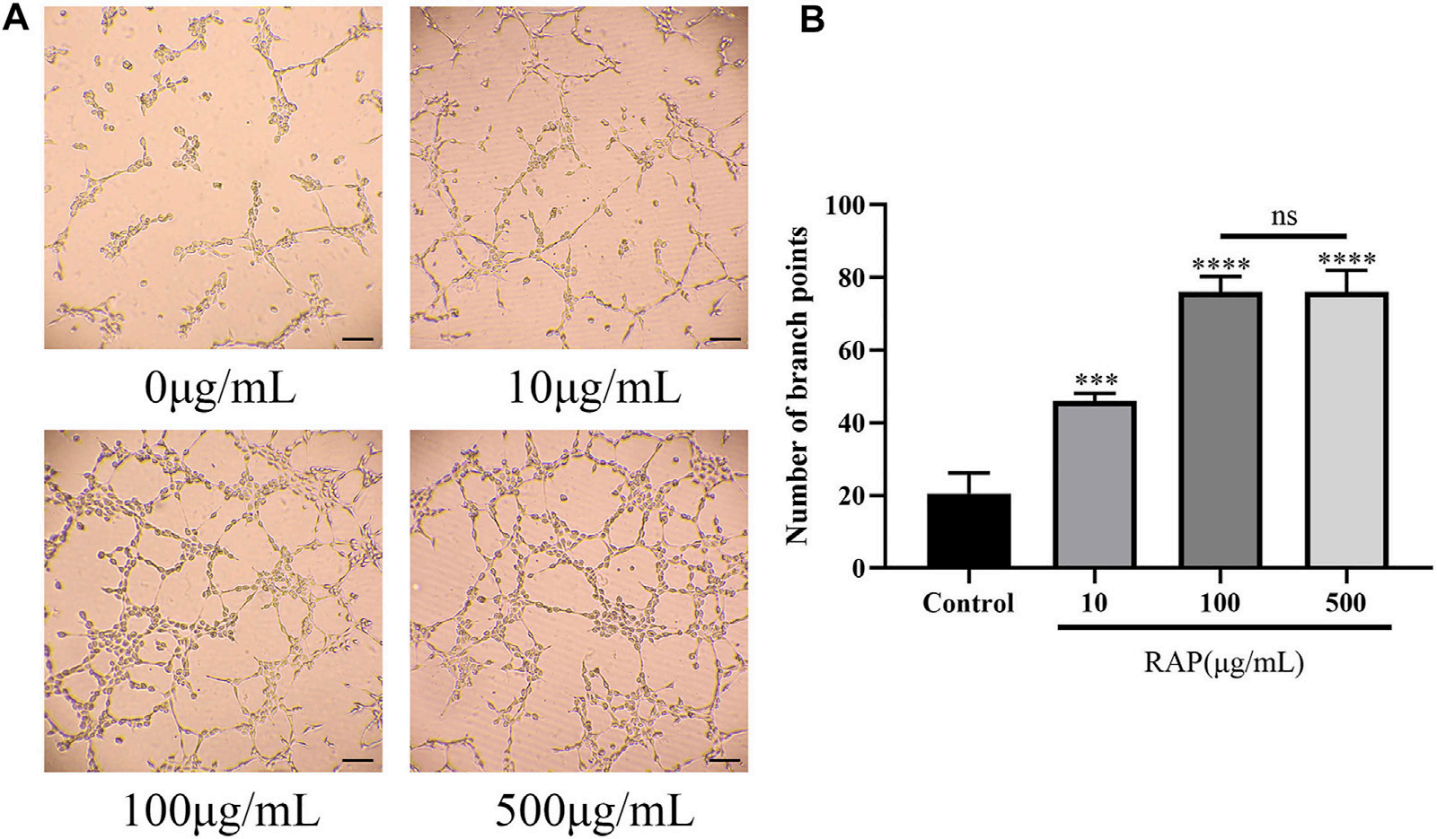
RAP promoted EC tube formation **(A)** Representative images of tube formation after EC treatment with different concentrations of RAP for 4 h (100× amplification, scale bar = 100 μm); **(B)** Number of branch points of ECs treated with different concentrations of RAP for 4 h. Data are presented as means ± SD. ***p < 0.001, ****p < 0.0001 compared with the control group.

We examined EC tube formation after treatment with different RAP concentrations using capillary network formation assay. At RAP concentrations ≤100 μg/ml, EC tube formation capacity gradually increased in a RAP concentration-dependent manner. However, there were no significant differences in EC tube formation capacity at RAP concentrations between 100 and 500 μg/ml (Figure 2, *p* < 0.05). These results showed that RAP significantly increased EC tube formation capacity at concentrations ≤100 μg/ml.

### RAP Promoted Angiogenesis by Activating the AKT/eNOS Signaling Pathway *in vitro*

**FIGURE 3:**
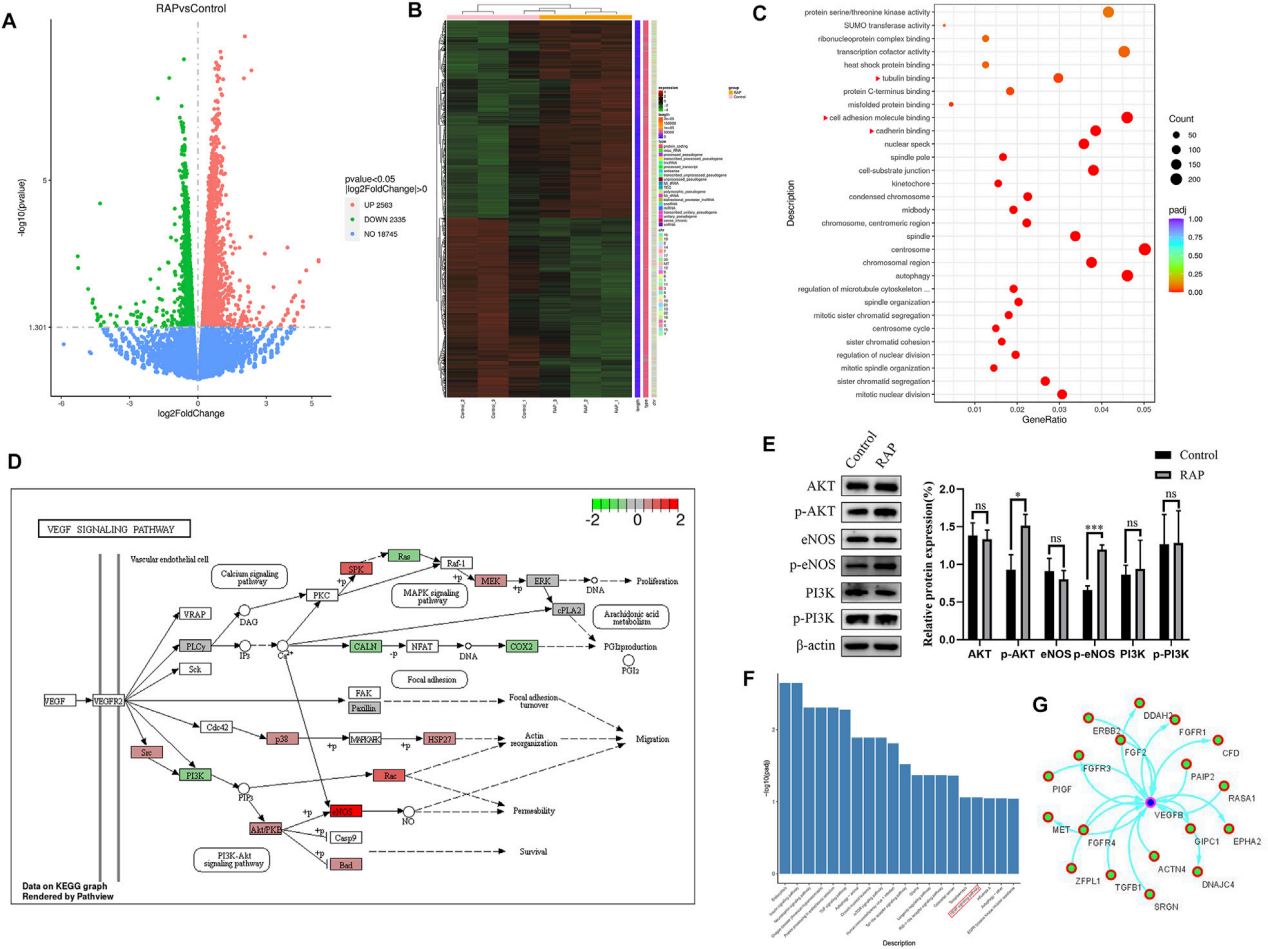
RAP promoted angiogenesis by activating the AKT/eNOS signaling pathway *in vitro* **(A)** The volcano plot of DEGs; **(B)** The heat map of DEGs; **(C)** The GO function analysis, the red triangle indicates the enrichment associated with angiogenesis; **(D)** DEGs are enriched in the VEGF signaling pathway (www.kegg.jp); **(E)** Representative western blotting and statistical analysis of AKT, p-AKT, eNOS, p-eNOS, PI3K, p-PI3K; **(F)** The KEGG pathway analysis, the red box represents the VEGF signaling pathway; **(G)** The PPI network analysis of proteins encoded by DEGs and VEGFB. Data are presented as means ± SD. *p < 0.05, ***p < 0.001.

We revealed the mechanism of RAP-promoted angiogenesis by RNA-seq. We compared the RAP treatment group with the control group and identified a total of 4,898 DEGs (padj ≤0.05 and |log2 (foldchange)| ≥ 0). In the RAP treatment group, 2,563 genes were upregulated and 2,335 genes were downregulated, respectively (Figures 3A,B). GO enrichment revealed a large number of DEGs that are closely associated with angiogenesis, such as cadherin binding, cell adhesion molecule binding, and tubulin binding (Figure 3C). We found a large number of DEGs enriched in VEGF signaling pathway by KEGG database analysis (Figures 3D,F). Then, we found that RAP significantly promoted the expression of p-AKT and p-eNOS by western blot. However, AKT and eNOS were not significantly changed. PI3K, as one of the upstream molecules of AKT/eNOS signaling pathway, was also verified. Interestingly, PI3K was not activated. In future work, we will focus on identifying other important molecules of AKT/eNOS signaling pathway (Figure 3E). Finally, we used the MCODE plug-in in Cytoscape software to analyze the PPI network and revealed the relationship between proteins encoded by DEGs with VEGFB (Figure 3G). Taken together, RAP may accelerate angiogenesis by activating the AKT/eNOS signaling pathway.

Our results showed that RAP increased EC migration and tube formation in a concentration-dependent manner. Next, we explored the effect of RAP at 100 μg/ml on peripheral nerve regeneration *in vivo*.

### RAP Promoted Functional Recovery After PNI

**FIGURE 4:**
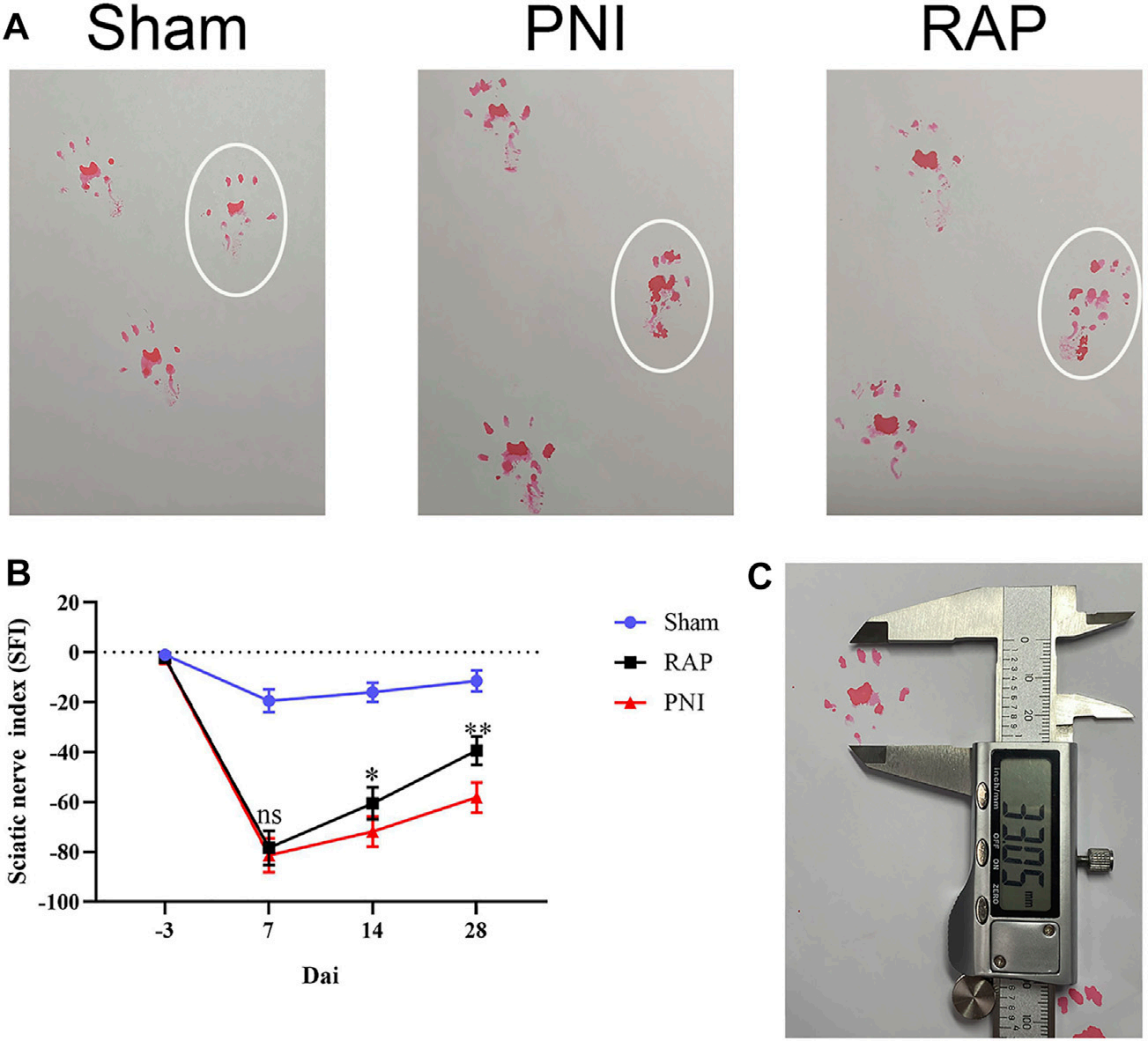
RAP promoted functional recovery after PNI **(A)** Representative images of footprints of each group of rats 28 days after PNI; white circle shows the footprints on the injured side; **(B)** Sciatic nerve function index (SFI) analysis was performed for each group on days 7, 14, and 28; **(C)** Representative image of the footprint measurement method. Data are presented as means ± SD. *p < 0.05, **p < 0.01.

We assessed whether RAP had a therapeutic effect in a PNI animal model. Nerve functional recovery after PNI was assessed weekly using walking track analysis. The SFI improved gradually over time, but no significant differences were found between the two nerve crush groups before day 7. After 14 days of treatment, the SFI values of the RAP-treatment group were higher than those in the PNI group but lower than the sham group. This difference was more pronounced at day 28 post-treatment (Figure 4, *p* < 0.05). In summary, RAP treatment was beneficial for functional recovery after sciatic nerve crush injury.

### RAP Promoted Axonal Regeneration After PNI

**FIGURE 5:**
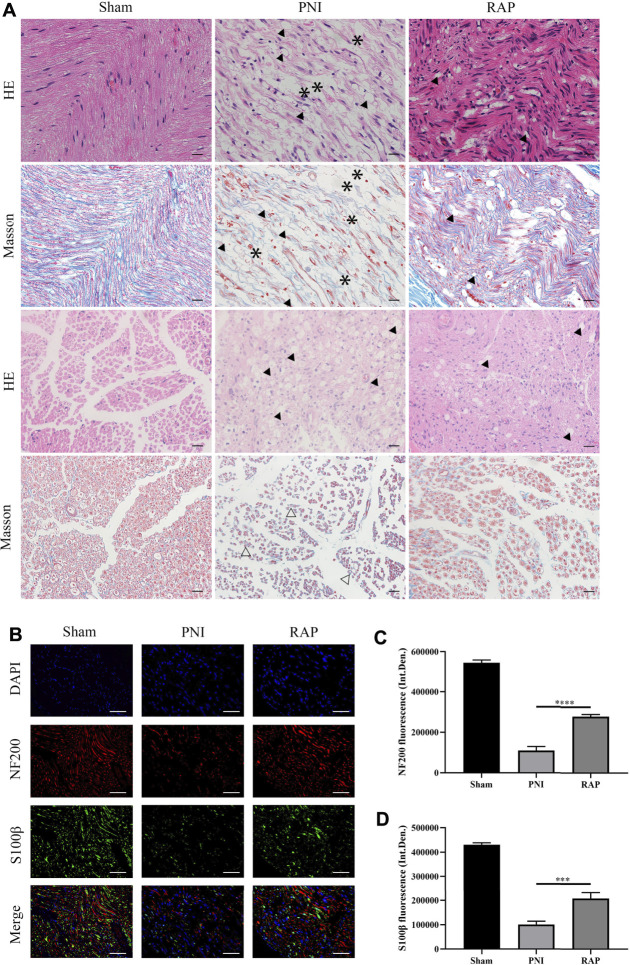
RAP promoted axonal regeneration **(A)** Representative images of HE and Masson staining sciatic nerve longitudinal and transverse sections in each group 28 days after PNI (400× amplification, scale bar = 20 μm), arrows show vacuolization, asterisks show interrupted nerves, hollow arrows show atrophic nerves; **(B)** Representative images of double immunofluorescence staining for NF200 (red) and S100β (green) of sciatic nerve longitudinal sections in each group 28 days after PNI. Nuclei are stained with DAPI (blue) (400× amplification, scale bar = 50 μm); **(C)** Statistical analysis of fluorescence integrated density of NF200; **(D)** Statistical analysis of fluorescence integrated density of S100β. Data are presented as means ± SD. ***p < 0.001, ****p < 0.0001.

To evaluate the morphology of sciatic nerves and to assess the histological changes in the injured nerve fibers, HE and Masson staining were performed on longitudinal and transverse sections of nerves after 28 days of treatment. The results showed that nerve fibers in the PNI group were scarce, disordered, vacuolated, and decreased in diameter. However, in PNI animals treated with RAP, nerve fibers were arranged tightly and orderly, and edema and vacuolization were greatly reduced (Figure 5A). We examined the effect of RAP on axonal regeneration and proliferation of Schwann cells 28 days after PNI by fluorescence immunohistochemistry staining to detect NF200 and S100β. Compared with the PNI group, NF200- and S100β-associated fluorescence intensity was significantly higher in the RAP treatment group (Figures 5B–D, *p* < 0.05). In summary, RAP could significantly improve abnormal nerve fiber morphology and promote axonal regeneration following PNI.

### RAP Promoted Remyelination After PNI

**FIGURE 6:**
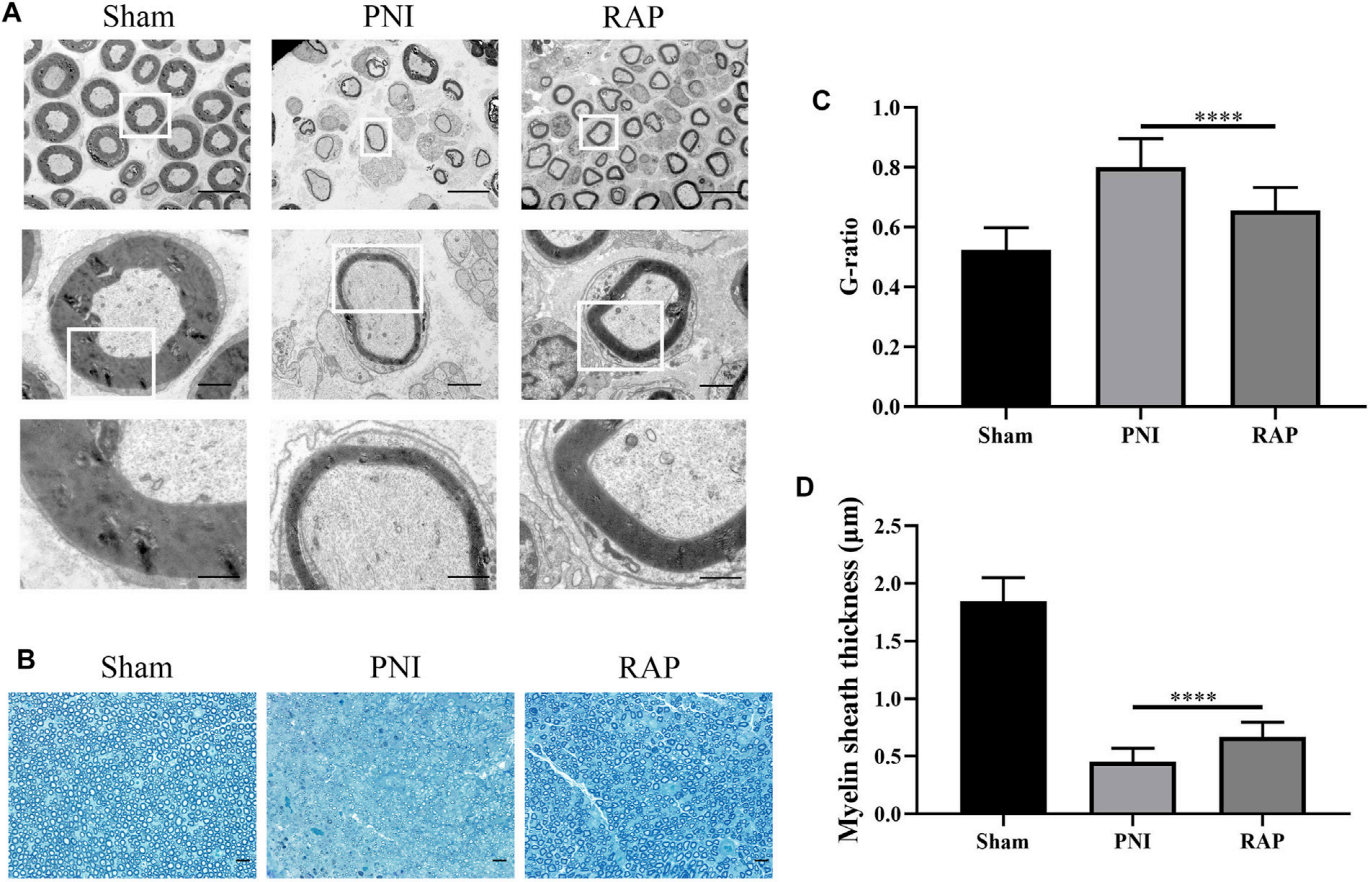
RAP promoted remyelination after PNI **(A)** Representative TEM images of sciatic nerve transverse sections in each group 28 days after PNI (1000×, 4,000×, 10000× amplification, scale bars = 10 μm, 2 μm, or 1 μm); **(B)** Representative images of TB staining of sciatic nerve transverse sections in each group 28 days after PNI (400× amplification, scale bar = 20 μm); **(C)** Statistical analysis of G-ratio; **(D)** Statistical analysis of myelin sheath thickness. Data are presented as means ± SD, ****p < 0.0001.

To investigate the effect of RAP on myelin regeneration after PNI, we observed the histological changes in regenerated myelin in each group by transmission electron microscopy and toluidine blue staining after 28 days of treatment (Figures 6A,B). Myelin thickness was reduced in the PNI group. However, these abnormalities improved significantly after RAP treatment. Moreover, statistical analysis showed that the G-ratio in the RAP treatment group was lower than that in the PNI group and the myelin thickness was significantly greater than that in the PNI group (Figures 6C,D, *p* < 0.05). Taken together, these results suggested that RAP could promote myelin remodeling after PNI.

### RAP Delayed Gastrocnemius Muscle Atrophy

**FIGURE 7:**
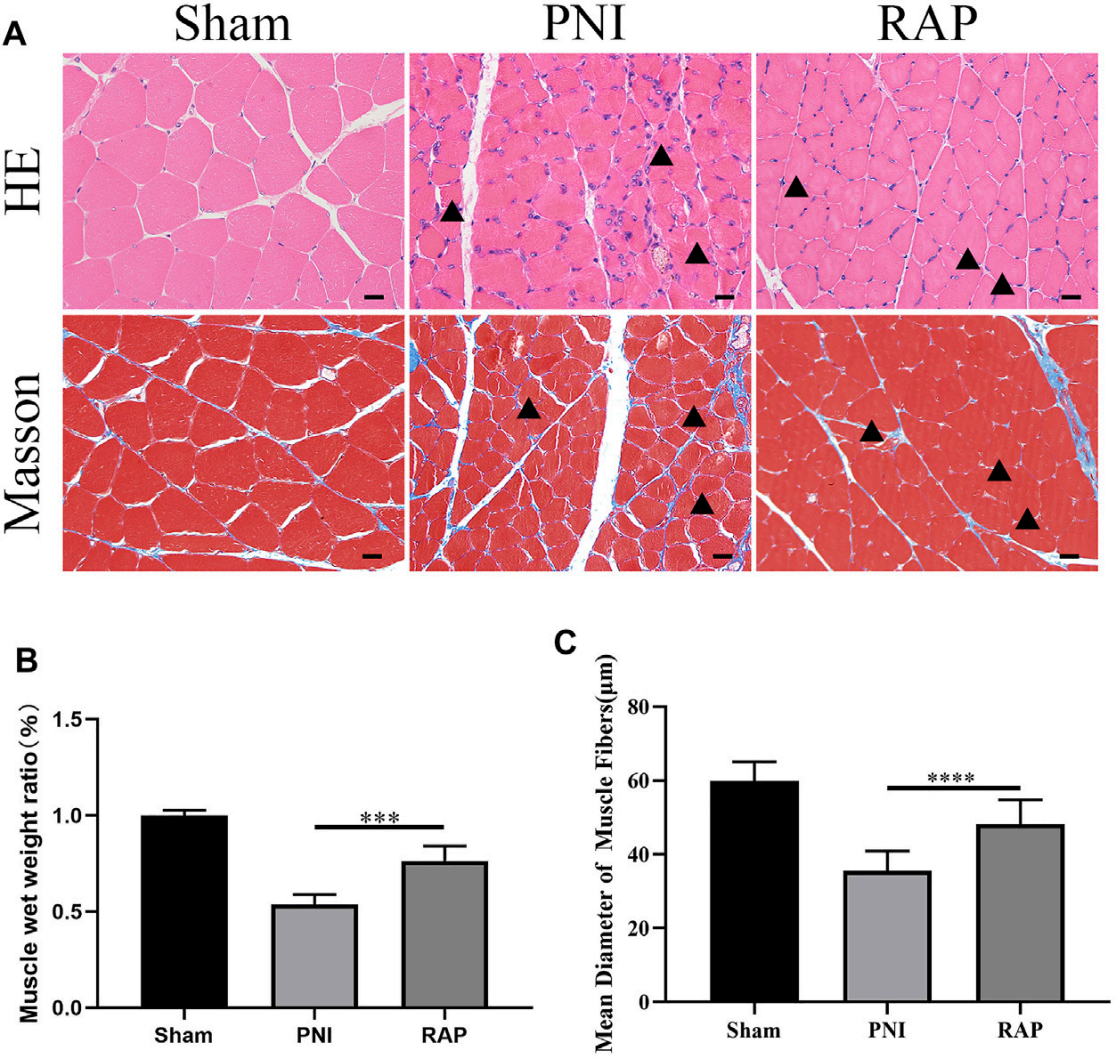
RAP delayed gastrocnemius muscle atrophy after PNI **(A)** Representative HE and Masson staining images of gastrocnemius muscle transverse sections in each group 28 days after PNI (400× amplification, scale bar = 20 μm), arrows show atrophied muscle fiber; **(B)** Statistical analysis of muscle wet weight ratio; **(C)** Statistical analysis of muscle fiber mean diameter. Data are presented as means ± SD. ***p < 0.001, ****p < 0.0001.

We evaluated gastrocnemius muscle morphology by HE and Masson staining 28 days after treatment. The arrows indicated atrophied muscle fibers, muscle atrophy existed in both RAP treatment group and PNI group, but the severity of RAP treatment was less than that of PNI group (Figure 7A). The muscle wet weight ratio was higher in the RAP treatment group than in the PNI group (Figure 7B, *p* < 0.05). In addition, compared to the PNI group, the mean diameter of muscle fibers was significantly larger in the RAP treatment group (Figure 7C, *p* < 0.05). In summary, our results confirmed that RAP could delay gastrocnemius atrophy after PNI.

### RAP Accelerated Angiogenesis of the Sciatic Nerve Through Activation of the AKT/eNOS Signaling Pathway

**FIGURE 8:**
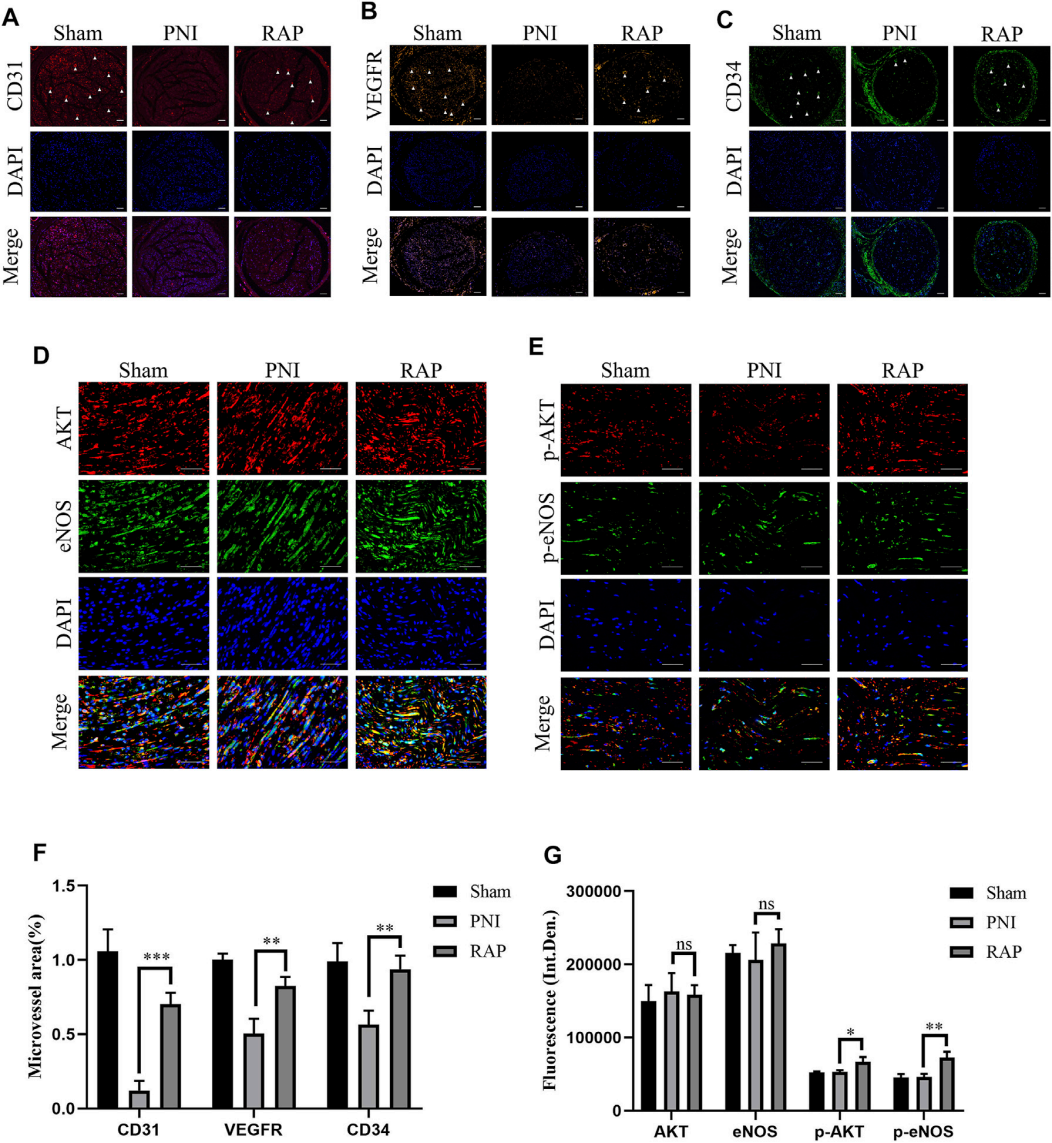
RAP Accelerated Angiogenesis of the Sciatic Nerve through activation of the AKT/eNOS signaling pathway **(A–C)** Representative images of immunofluorescence staining for CD31 (red), VEGFR (orange), CD34 (green) of sciatic nerve transverse sections in each group 28 days after PNI. Nuclei are stained with DAPI (blue) (100× amplification, scale bar = 100 μm), arrows show neovascularization; **(D)** Representative images of double immunofluorescence co-localization staining for AKT (red) and eNOS (green) of sciatic nerve longitudinal sections in each group 28 days after PNI. Nuclei are stained with DAPI (blue) (400× amplification, scale bar = 50 μm); **(E)** Representative images of double immunofluorescence staining for p-AKT (red) and p-eNOS (green) of sciatic nerve longitudinal sections in each group 28 days after PNI. Nuclei are stained with DAPI (blue) (400× amplification, scale bar = 50 μm) **(F)** Statistical analysis of Microvessel area **(G)** Statistical analysis of fluorescence integrated density of AKT, eNOS, p-AKT, and p-eNOS. Data are presented as means ± SD. *p < 0.05**, *p* < 0.001,***p < 0.0005.

To investigate the angiogenesis of sciatic nerves, we used fluorescence microscopy to detect the specific markers of angiogenesis marker CD31, CD34, and VEGFR at 28 days after treatment. Our results showed that the microvessel area was significantly higher in the RAP treatment group compared to the PNI group (Figures 8A–C,F, *p* < 0.05). Subsequently, we detected the AKT/eNOS axis *in vivo* by immunofluorescence co-localization. We found no significant difference in the fluorescence intensity of AKT and eNOS between the PNI and RAP treatment groups (Figures 8D,G, *p* > 0.05). However, the fluorescence intensity of p-AKT and p-eNOS was significantly stronger in the RAP treatment group (Figures 8E,G, *p* < 0.05). These results demonstrated that RAP could effectively promote peripheral nerve angiogenesis through activation of the AKT/eNOS signaling pathway.

**FIGURE 9:**
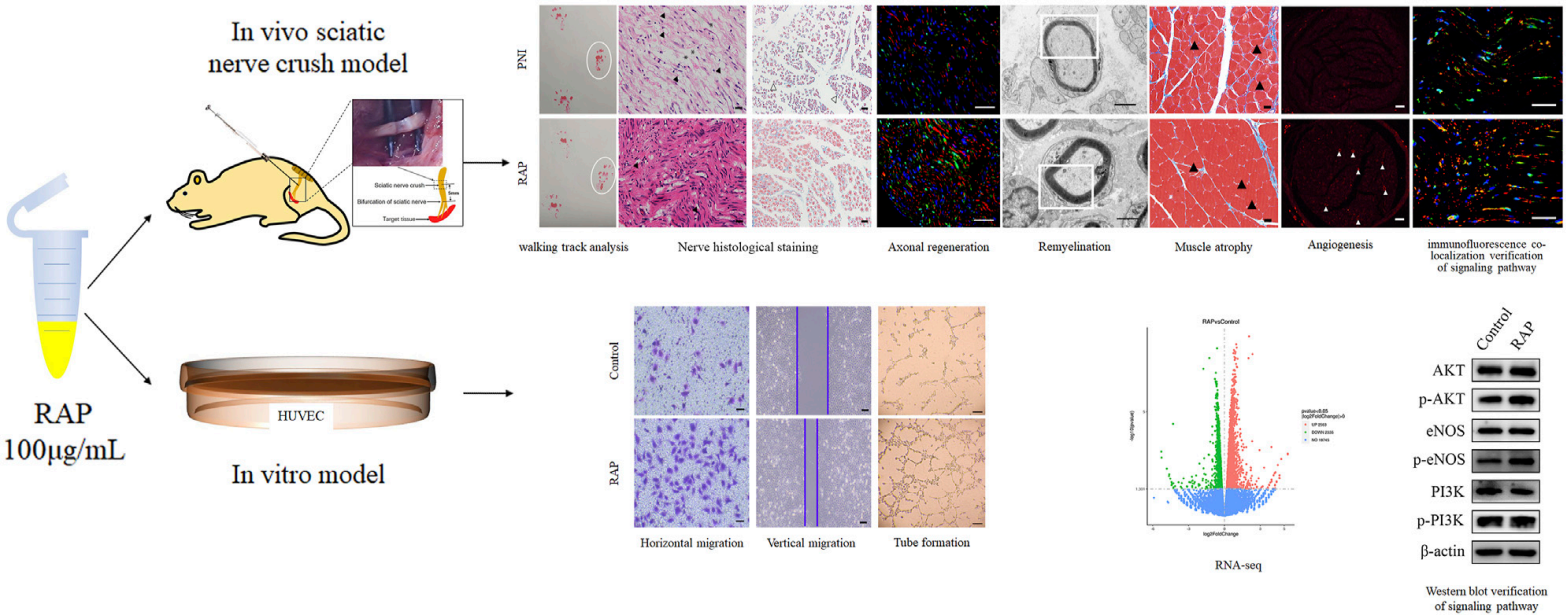
Schematic diagram of the study. *In vitro*, RAP promoted EC migration and tube formation. RNA-seq and WB revealed that RAP may accelerate angiogenesis by activating AKT/eNOS signal pathway. *In vivo*, RAP promoted functional recovery, delayed target organ atrophy, improved neuropathological morphology and remyelination after PNI, and could accelerate angiogenesis by activating AKT/eNOS signaling pathway.

**A schematic** diagram of this study was provided for reference (Figure 9).

## Discussion

RAP was commonly used for anti-tumor, immunomodulatory, anti-inflammatory, and antioxidant purposes. However, in the present study, we focused on the role of RAP for peripheral nerve regeneration and tried to reveal the underlying mechanisms for its effect. The new findings of RAP regulation of PNI are as follows: 1) RAP has a strong neuroprotective effect on the PNS, as demonstrated by its ability to promote functional nerve recovery, delay target organ atrophy, and promote the orderly regeneration and remyelination of axons; 2) The neuroprotective effect of RAP may be related to accelerated angiogenesis at the injury site, thereby directing the extension of new axons; 3) RAP notably accelerates angiogenesis by activating AKT/eNOS signaling pathway.

It is well known that neurotrophic growth factors play an essential role in PNI repair. Each neurotrophic growth factor may promote nerve regeneration through different signaling pathways and optimal concentrations (Faroni et al., 2015). Recent studies have shown that nerve growth factor (NGF) expression was upregulated after PNI, and it induced neural protrusion growth by binding to Trk receptors and activating the Ras-extracellular signal-regulated kinase (ERK) pathway (Manoukian et al., 2020). It has been shown that brain-derived neurotrophic factor (BDNF) promotes nerve regeneration by activating the Janus kinase-signal transducer and activator of transcription (JAK/STAT) pathway in Schwann cells (Lin et al., 2016). Tajdaran et al. used a drug delivery system with poly (lactic-co-glycolic acid) microspheres to deliver exogenous glial cell line-derived neurotrophic factor (GDNF), resulting in a significant increase in the number of motor and sensory nerves after injury (Tajdaran et al., 2016). However, due to its specific pharmacokinetic and physicochemical properties, NGF had poor stability, a short half-life with time-dependent limitations, and its production was expensive (Pfister et al., 2007). Therefore, there is an urgent need for a new drug for peripheral nerve regeneration repair. We hope to provide a new strategy for PNI repair using RAP.

In this study, we used intraneural RAP injection in a rat sciatic nerve crush model to explore the effect of RAP on peripheral nerve regeneration. We found that RAP treatment significantly improved the speed and extent of functional recovery, as shown by SFI and footprint analysis (Figure 4). In addition, we observed significant improvements in histological and morphological recovery with RAP-treatment by histological and immunofluorescence staining, as evidenced by axonal regeneration and reduced neurodegeneration (Figure 5). It has been shown that axonal remyelination facilitates the recovery of neurological function (Jessen and Mirsky, 2005). To address this issue, we examined myelin status by TB staining and TEM. The results showed that RAP promoted axonal remyelination. In addition, the G-ratio and myelin thickness were significantly increased in the RAP treatment group compared with the PNI group (Figure 6). We found that RAP could delay the atrophy of target organs after PNI by using HE and Masson staining, muscle fiber diameter measurements, and analysis of gastrocnemius muscle wet weight (Figure 7). These analyses suggested that RAP can exert a neuroprotective effect to improve the recovery of neural structure and function after PNI. However, the underlying mechanisms through which RAP modulates the recovery of peripheral nerve function remain poorly understood.

Previous studies had shown that vascular regeneration preceded axonal regeneration (Cattin et al., 2015). Induction of vascular regeneration had a positive effect on nerve regeneration (Zor et al., 2014). Ouyang et al. demonstrated that dual delivery of plasmid-encoded vascular endothelial growth factor and NGF as gene therapy could promote sciatic nerve regeneration (Fang et al., 2020). Intraneural vascularization was a major factor in the neural regeneration microenvironment (Qian et al., 2021). Based on these studies, we hypothesized that RAP could accelerate early angiogenesis to promote functional recovery after PNI. To test this hypothesis, we investigated the effect of RAP on angiogenesis by using EC migration and capillary network formation assay. We observed that RAP significantly enhanced EC migration and tube formation (Figure 1 and Figure 2). Angiogenesis was regulated by multiple cascades of signaling, including the VEGF signaling pathway, Notch, KLF2, and YAP signaling pathways (Dekker et al., 2005; Tammela et al., 2011; Paauwe et al., 2013; Nakajima et al., 2017). Among them, the VEGF signaling pathway was regarded as a key regulatory mechanism in vascular regeneration (Gucciardo et al., 2018). We found a large number of differential gene enrichment in key proteins AKT and eNOS in the VEGF pathway by RNA-seq, previous studies have shown that angiogenesis in peripheral nerves can be promoted by activating the AKT/eNOS axis (Qian et al., 2018). We verified that RAP significantly promoted the expression of p-AKT and p-eNOS and activated the AKT/eNOS signaling pathway by western blot. We suggested that the role of RAP in angiogenesis may be associated with AKT/eNOS signaling pathway activation (Figure 3). In addition, we found that RAP significantly promoted the expression of the specific markers of angiogenesis marker CD31, CD34, and VEGFR. And the results showed that RAP significantly activated the AKT/eNOS axis by immunofluorescence co-localization assay *in vivo*. (Figure 8). Collectively, these results suggested that RAP has a powerful ability to accelerate angiogenesis and provides clues to neural regeneration.

## Conclusion

To sum up, we report for the first time that RAP promoted functional recovery after PNI, delayed target organ atrophy, and mediates regeneration and remyelination of axons. Furthermore, the positive effect of RAP on nerve regeneration was associated with angiogenesis that may be associated with activation of the AKT/eNOS signaling pathway. Overall, our results suggested that RAP may be a potentially effective therapeutic agent for PNI repair.

## Data Availability

The datasets presented in this study can be found in online repositories. The names of the repository/repositories and accession number(s) can be found below: https://www.ncbi.nlm.nih.gov/geo; GSE193452.
